# Artificial Intelligence in Head and Neck Surgical Oncology: A State-of-the-Art Review

**DOI:** 10.3390/jcm15072767

**Published:** 2026-04-06

**Authors:** Steven X. Chen, Maria Feucht, Aditya Bhatt, Janice L. Farlow

**Affiliations:** Department of Otolaryngology—Head and Neck Surgery, Indiana University School of Medicine, Indiana University, Indianapolis, IN 46202, USA

**Keywords:** artificial intelligence, machine learning, deep learning, large language model, computer vision, augmented reality, head and neck, surgery, otorhinolaryngology, oncology

## Abstract

Artificial intelligence (AI) is rapidly reshaping head and neck surgical oncology by augmenting decision-making across the full perioperative continuum. This state-of-the-art review aims to provide head and neck surgical oncologists with a conceptual framework for understanding and critically appraising AI tools entering clinical practice, summarizing how machine learning, deep learning, and generative AI are being integrated into contemporary surgical workflows. Preoperative applications include detection of occult nodal metastasis and extranodal extension. Intraoperative innovations include augmented reality-assisted navigation, real-time margin assessment, and improving visual clarity and tissue handling for robotic platforms. Postoperatively, AI can predict complications like free flap failure and oncologic outcomes. Large language models are being operationalized for clinician-facing applications such as documentation and inbox support, as well as patient-facing education. Despite promising results, broad clinical deployment remains limited by concerns about privacy, validation, reliability, safety, and ethics. Widespread adoption will require prospective clinical trials, robust governance, and human-centered workflows that ensure AI remains a safe, assistive copilot.

## 1. Introduction

In recent years, artificial intelligence (AI) has emerged as a promising tool to improve diagnostics, treatment planning, and outcomes in medicine. In head and neck surgical oncology, AI applications remain in relatively early stages but are rapidly expanding. AI techniques are being explored across the continuum of care from preoperative diagnostic imaging and surgical planning to intraoperative guidance and decision support, and postoperative outcome prediction and surveillance. While AI has previously been largely within the purview of computational scientists, a fluency with and critical appraisal of increasing applications of AI will soon be required of clinicians. This review highlights relevant key AI concepts, the current state of AI in head and neck cancer surgery, challenges and considerations, and future directions. The primary objective of this review is to equip head and neck surgical oncologists with a conceptual framework for understanding, evaluating, and responsibly integrating AI into clinical practice. It highlights both the current promise and the meaningful barriers that remain before widespread adoption is appropriate.

## 2. Methods

This state-of-the-art narrative review was conducted to synthesize the current literature on AI applications across the perioperative continuum in head and neck surgical oncology. A structured literature search was performed in PubMed/MEDLINE and Google Scholar using the following Medical Subject Headings (MeSH) and keyword terms.

AI Terms:

“Artificial Intelligence” OR “Machine Learning” OR “Deep Learning” OR “Neural Networks, Computer” OR “Natural Language Processing” OR “Computer Vision” OR “Convolutional Neural Network” OR “Random Forest” OR “Support Vector Machine” OR “Large Language Model” OR “Robotics” OR “Radiomics” OR “Digital Pathology”.

Head & Neck Surgery Terms:

“Head and Neck Neoplasms” OR “Head and Neck Surgery” OR “Otolaryngology” OR “Laryngectomy” OR “Thyroidectomy” OR “Parotidectomy” OR “Neck Dissection” OR “Oral Cavity” OR “Oropharyngeal Neoplasms” OR “Laryngeal Neoplasms” OR “Salivary Gland Neoplasms” OR “Skull Base Surgery” OR “Free Flap”.

Searches were limited to articles published in English between January 2015 and March 2026, with additional seminal references included irrespective of publication date when deemed foundational to a given topic. Articles were screened for relevance to the clinical application of AI in head and neck surgical oncology, including both primary research studies and systematic reviews. Studies were selected based on clinical relevance, methodological quality, novelty, and topical coverage across the perioperative workflow. Given the rapid evolution of this field, preprints and recently published peer-reviewed articles were included when they represented significant advances.

A narrative review format was selected as most appropriate for this topic for several reasons. First, the field of AI in head and neck surgical oncology is heterogeneous and rapidly evolving, spanning diverse methodologies, anatomic subsites, and clinical contexts that resist the uniform eligibility criteria and quantitative synthesis required by systematic or scoping review frameworks. Second, the primary aim of this review is conceptual and educational: to provide surgeons with an accessible framework for understanding and critically appraising a broad technology landscape rather than to answer a narrow, pre-specified clinical question amenable to meta-analysis. Third, the existing literature is insufficiently standardized in outcome reporting to permit meaningful pooled estimates. A narrative approach therefore allows the authors to integrate evidence across heterogeneous study types, highlight methodological strengths and limitations, and draw clinically oriented conclusions that would be obscured by the constraints of more formal review methodologies.

Because of the narrative review format, formal PRISMA methodology and meta-analysis were not employed. Instead, the authors synthesized findings thematically across preoperative, intraoperative, and postoperative domains, with attention to study design, validation approach, and clinical applicability. Key performance metrics (e.g., AUC, sensitivity, specificity) are summarized where reported.

## 3. Principles of Artificial Intelligence in Surgery

**Figure 1:**
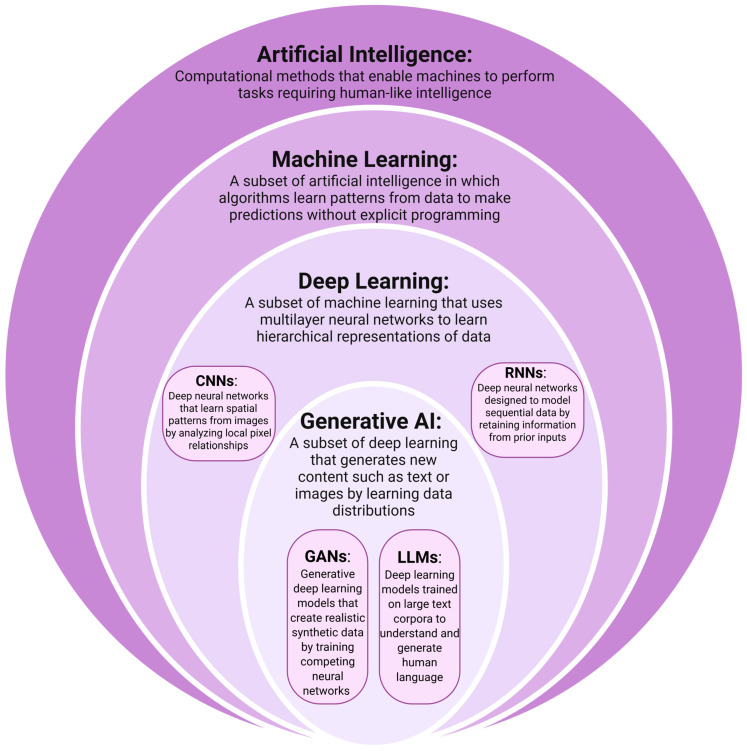
Hierarchical Framework of Artificial Intelligence. The hierarchy progresses from broad theoretical frameworks to specific architectural implementations. Artificial Intelligence (AI): The overarching domain encompassing computational systems designed to emulate human cognitive functions, such as reasoning, problem-solving, and decision-making in clinical environments. Machine Learning (ML): A critical subset of AI focusing on the development of algorithms that improve performance through experience. In medicine, ML is used to identify patterns in structured data (e.g., electronic health records) to facilitate predictive analytics without being explicitly programmed for every scenario. Deep Learning (DL): A specialized branch of ML utilizing multilayered neural networks to process complex, high-dimensional data. Convolutional Neural Networks (CNNs): Optimized for spatial data, primarily used for automated medical imaging analysis. Recurrent Neural Networks (RNNs): Designed for sequential data, such as real-time physiological monitoring or longitudinal patient history. Generative AI: A modern subset of deep learning capable of creating novel data outputs. Generative Adversarial Networks (GANs): Employed for creating synthetic medical data or enhancing image resolution. Large Language Models (LLMs): Trained on vast corpora of text to assist in natural language processing (NLP) tasks, such as automated clinical documentation or medical question-answering.

**Figure 2:**
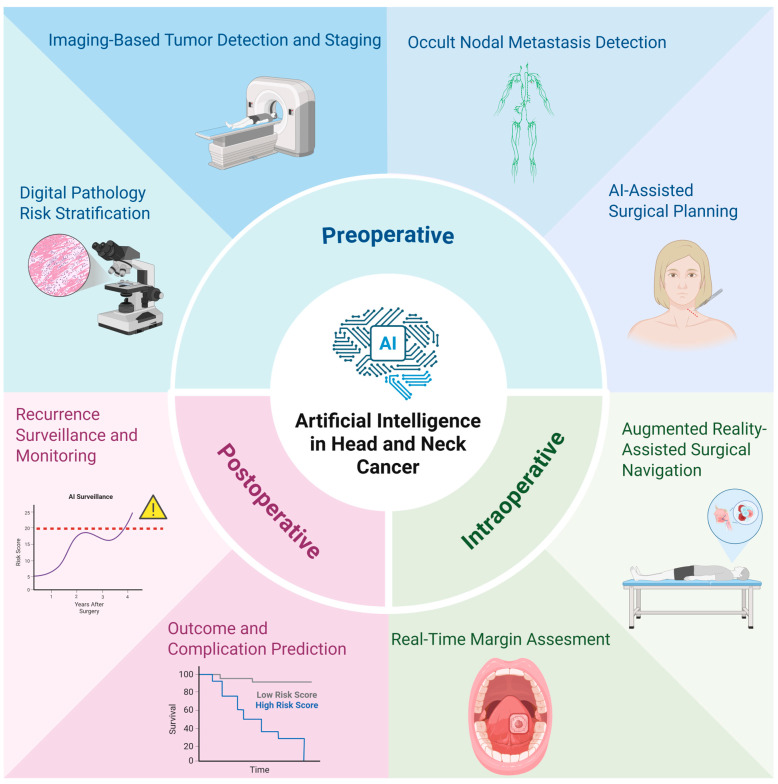
The Artificial Intelligence (AI) Continuum in Head and Neck Surgical Oncology. This schematic illustrates potential AI applications across the preoperative, intraoperative, and postoperative phases of care. Preoperative Phase: AI applications facilitate precision diagnostics through digital pathology for automated risk stratification and deep-learning-based surgical planning, optimizing patient selection and reconstructive mapping. Intraoperative Phase: Real-time assistance is provided via augmented reality (AR)-assisted navigation, which overlays genomic or radiologic data onto the surgical field, and AI-driven margin assessment (e.g., via hyperspectral imaging or optical coherence tomography) to ensure complete oncologic resection. Postoperative Phase: Predictive analytics utilize longitudinal data to model survival outcomes and complication risks, while computer-aided surveillance systems monitor for early recurrence, enabling personalized follow-up and timely intervention.

AI is a broad category of computational methods and technology designed to simulate human learning, comprehension, problem solving, decision making, creativity and autonomy (Figure 1). Within surgical oncology, it is best thought of as a set of methods that learn patterns from data to support decisions and workflows across the perioperative timeline, including preoperative (risk stratification, imaging interpretation, surgical planning), intraoperative (navigation, image guidance, margin assessment support), and postoperative (complication prediction, surveillance, patient-facing education) phases (Figure 2, Table 1) [1]. Input data is often multimodal, including cross-sectional imaging, endoscopy video, digital pathology, laboratory results, operative reports, tumor board notes, and longitudinal outcomes.

Machine Learning (ML) refers broadly to algorithms that learn patterns from data without explicitly programmed rules [12]. Traditional ML often requires manual “feature engineering,” where domain experts select which variables the model will use. Deep Learning (DL), a subset of ML, eliminates the need for manual feature extraction, and is thus the engine behind the most advanced applications, particularly in image analysis and computer vision [13]. Convolutional Neural Networks (CNNs) are the workhorses of medical image analysis, designed to process grid-like data such as computed tomography slices [14,15].

The newest frontier is Generative AI, which include Large Language Models (LLMs) and Generative Adversarial Networks (GANs). Unlike discriminative models which classify data, generative models create new data. LLMs are being explored for automated note generation and summarizing patient histories [16,17]. Additionally, GANs can impute synthetic medical images, potentially filling in missing data in corrupted or low-resolution images to enhance diagnostic robustness.

**Figure 3:**
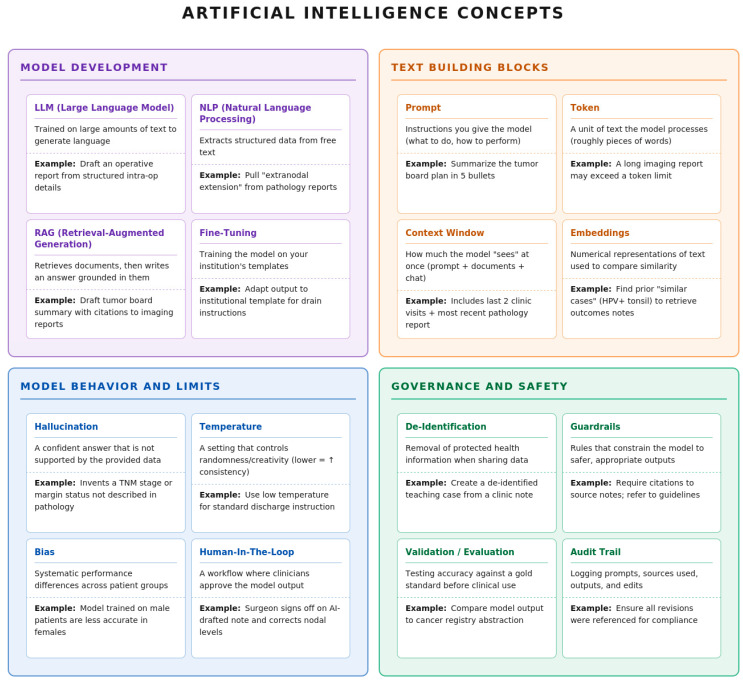
Glossary of Artificial Intelligence Terminology. The glossary is organized into four functional domains. (1) Model Development: Describes technical methodologies for processing surgical data. (2) Text Building Blocks: Defines the fundamental units of model interaction. (3) Model Behavior and Limits: Outlines critical safety and performance parameters. (4) Governance and Safety: Details the ethical and regulatory requirements for AI integration.

A variety of terms are used commonly for AI applications that may be less familiar to surgical oncologists (Figure 3). A prompt is the instruction and constraints an LLM receives. Temperature controls how deterministic or variable the output is (lower is more precise, higher is more creative). Natural language processing (NLP) broadly refers to extracting/structuring information from text (e.g., margin status, nodal levels), while LLMs can also generate text (drafting summaries, letters, discharge instructions). The major safety principle here is acknowledging and engineering around hallucinations, or statements not supported by source data. Healthcare-focused evaluations show hallucinations and omissions can occur in medical summarization and extraction, motivating structured evaluation frameworks and clinician review [18].

To reduce hallucination and improve traceability, many teams use embeddings (numeric representations of text meaning) plus retrieval-augmented generation (RAG) to retrieve the most relevant source snippets (e.g., pathology result, radiology impression, operative report) and feed them into the model so the output is grounded in known documents. This enables “answer with citations to the chart,” which is attractive for tumor board summaries and staging extraction. Systematic reviews show RAG often improves factuality but requires standardized evaluation and careful choice of retrieval methods and reference sources [19,20]. Relatedly, fine-tuning (additional training on smaller, task-specific datasets) can match output to institutional-specific terminology, templates, and documentation patterns [21].

Safe deployment in surgical oncology hinges on governance. Protected health information (PHI) should be safeguarded with de-identification or controlled environments. Guardrails on what the system may and may not do should be defined. A human-in-the-loop where a human reviewer can verify and approve the AI output can also be deployed for clinical sign-off. Audit trails of sources, outputs, and edits should be carefully maintained. Finally, before scaling, validation of models against gold standards should be tested. These ideas map cleanly onto major guidance and frameworks: the United States Health and Human Services explains HIPAA de-identification pathways (Safe Harbor and Expert Determination), National Institute of Standards and Technology’s AI Risk Management Framework organizes trustworthy AI around governance and measurable risk controls, the World Health Organization emphasizes ethics/human rights and accountability in AI for health, and the Food and Drug Administration/International Medical Device Regulators Forum Good Machine Learning Practice principles stress lifecycle thinking for AI/ML medical products, including safety and effectiveness considerations [22,23,24,25].

## 4. AI in the Perioperative Workflow

### 4.1. The Patient-Clinician Relationship

Beneficial AI systems integrated into head and neck surgical oncology should be designed as assistive copilots—incorporated into existing clinical systems, accessible and understandable by users, constrained by appropriate guardrails, with reliable and reproducible processes that are available for human review—so that speed and consistency improve without sacrificing accuracy, safety, or trust. In practice, that may mean clinician-facing language tools that structure a patient’s course and management (automated notes, concise history/timeline summaries, suggested orders and portal message replies) and patient-facing tools that convert the same plan into clear, accessible education (plain-language instructions, chat-based navigation), while preserving clinician accountability.

On the clinician-facing side, one of the most immediately practical applications of generative AI is documentation support via ambient listening (or ambient clinical intelligence) tools that draft a note for the clinician to review and sign. The clinician remains responsible for correctness, completeness, and clinical reasoning. A large multi-site survey study of ambient documentation technology (AI-drafted notes from clinician–patient conversations) suggested that such technology may reduce burnout and increase perceived well-being [11]. Because ambient tools often rely on audio capture, clinicians must be able to navigate patient communication and consent workflows: education, trust, and clear opt-out options [26].

Another clinician-facing use case is summarizing patient histories and compressing long charts into decision-ready narratives (e.g., pre-op risk summaries, tumor board note, treatment timeline, discharge summaries). One study demonstrated that hospital course summaries for discharge documentation written by an EHR-embedded LLM were rated as higher quality than physician-written ones [27]. This matters in complex longitudinal diseases like head and neck cancer, which often involves multifaceted data related to multimodality therapy, frequent recurrences, and multidisciplinary care.

A separate study investigating patient inbox support showed that an EHR-integrated, HIPAA-compliant LLM that drafted inbox replies led to improvement in burnout, although message time metrics did not meaningfully change [28]. Another patient-facing investigation that had survey participants rate satisfaction with AI versus clinician responses to questions found higher satisfaction scores for AI responses [29]. In practice, this could translate into faster, more consistent first-draft answers for common post-op questions (wound/drain care, diet/swallow precautions, tracheostomy/laryngectomy care, expected radiation effects), with the clinical team ensuring safety, personalization, and appropriate escalation. One study showed LLM could transform inpatient discharge summaries into a more patient-friendly format with markedly improved readability/understandability, but also found safety concerns of omissions and inaccuracies, reinforcing that real-world use should include clinical review [30]. AI applications for post-operative patient education are attractive but must employ critical safety checks. Given that head and neck cancer surgeries often have complex discharge needs, significant issues can arise should erroneous information be communicated (e.g., airway management). Real-world use should consider clinical review and safety checks before giving content directly to patients.

Finally, patient-facing chatbots (education, navigation, symptom guidance, appointment preparation) can improve access and responsiveness, but practical deployment hinges on guardrails. Some evidence suggests chatbots can produce responses judged as high quality and empathetic [31]. At the same time, accuracy and sourcing are common failure points. Studies on chatbots for mental health show that there may be value for improving access and even symptom improvement, but also concerns for variable and event inappropriate responses as well [32,33]. A chatbot that can be perceived as a “medical authority” is risky without retrieval, citation, and escalation logic. Keeping a degree of human-in-the-loop supervision is a key safety design decision.

### 4.2. Preoperative Applications

The accessibility and accuracy of diagnosis, staging, and treatment planning dictate the entire treatment trajectory. A powerful early research area of AI in medicine centered on radiography, the interpretation of which can be complex, time-consuming, qualitative and subjective. AI algorithms can automatically segment primary head and neck tumors on scans with high accuracy comparable to expert radiologists [34]. AI systems have also shown expert-level sensitivity in detecting subtle lesions, aiding earlier diagnosis of cancers or precancerous conditions that might be missed on routine exam [35]. Beyond detection, radiomics can standardize image interpretation by transforming medical images into quantitative imaging features. These features can be used to develop ML and DL algorithms to improve risk stratification and predict outcomes in head and neck oncology. For head and neck oncology, where endoscopic images are commonly employed, AI can also be applied to these procedures to detect and classify cancers [36,37,38].

Additional algorithms have shown promise for automating staging in head and neck cancers based on multimodal DL pipelines [39]. Beyond this, AI is also being used to streamline clinical trial prescreening by translating inclusion and exclusion criteria into computable checks over both structured (e.g., laboratory values) and unstructured data (e.g., prose of clinical notes), reducing the manual chart-review burden that can meaningfully drive trial costs and delays [6,40]. This screening paradigm is particularly relevant to head and neck cancers, where trial eligibility often hinges on nuanced, frequently unstructured details such as tumor subsite and stage, prior surgery/radiation/systemic therapy, and biomarker status (p16 in oropharyngeal carcinoma).

In the realm of treatment, DL models can detect subtle perturbations in pixel intensity that correlate with metastatic infiltration, thus allowing more confident assessment and planning for nodal metastasis. A landmark study demonstrated an Area Under the Curve (AUC) of 0.9177 in a training cohort and 0.7205 in an external validation cohort for predicting occult metastasis [2]. This performance outperforms conventional criteria and suggests a future of AI-assisted decisions for elective neck, although the drop in performance across cohorts highlights the importance of external validation. It is worth noting that this performance gap between training data and external validation is characteristic of many current AI models in head and neck oncology and reflects the challenges of dataset heterogeneity, differences in imaging protocols, and institution-specific annotation practices. Until models demonstrate more consistent performance across independent datasets, their clinical utility for elective neck treatment decisions should be interpreted with caution.

Extranodal Extension (ENE) is a prognostic indicator that upstages a patient and often leads to aggressive multimodality treatment. Radiologists often struggle to detect ENE, with a 73% sensitivity in one meta-analysis [41,42]. DL algorithms trained on contrast-enhanced computed tomography scans have demonstrated superior accuracy in this task. Research indicates that AI-based algorithms applied to pretreatment scans can predict ENE with performance outperforming radiologists, identifying abstract prognostic features invisible to the naked eye [3,4]. Identifying ENE preoperatively permits more precise prognostication and may alter treatment planning (e.g., the use of upfront chemoradiation rather than surgery followed by adjuvant radiation with chemotherapy). Critically, while both referenced ENE studies demonstrate improved performance over radiologist assessment, neither has yet been prospectively integrated into a clinical decision pathway in a manner that demonstrably altered treatment outcomes. Their value in guiding the choice between upfront chemoradiation and surgery-first approaches remains to be established through prospective trials.

AI is increasingly enhancing histopathological assessment by improving diagnostic accuracy, reproducibility, and the extraction of prognostic features. In oropharyngeal squamous cell carcinoma, DL has been used to “virtually stain” pathology images between stain types such as hematoxylin and eosin to immunohistochemistry, allowing a tissue-preserving alternative to identifying new prognostic biomarkers [43]. AI-based image analysis has also been applied to predict human papillomavirus status in oropharyngeal squamous cell carcinoma directly from histology slides, offering a potential future rapid, cost-effective adjunct to molecular testing [5]. These approaches offer objective, reproducible pathology insights that may complement or even surpass conventional histologic evaluation.

Beyond diagnostic and staging applications, augmented reality (AR) is also emerging as a valuable tool in preoperative planning. Prior to surgery, AR-enabled platforms can integrate multimodal imaging data such as CT, MRI, and endoscopic video, to construct three-dimensional anatomical reconstructions that surgeons can explore and manipulate in a virtual environment [44]. This allows for rehearsal of complex resections, identification of critical structures at risk, and simulation of reconstructive options before the patient arrives in the operating room. AR-based preoperative planning has been explored across surgical specialties, where studies suggest it can improve spatial understanding of complex anatomy and may reduce intraoperative decision time [45,46,47]. In head and neck oncology specifically, preoperative AR visualization holds promise for procedures involving the skull base, parapharyngeal space, and proximity to the carotid artery, where anatomical complexity and the margin for error are highest. Integration of AI-based tumor segmentation with AR visualization further enables automatic overlay of predicted tumor boundaries and nodal basins onto three-dimensional surgical plans [48]. While the clinical maturity of these tools remains investigational and prospective outcome data are limited, they represent a logical upstream application of the same AR infrastructure being developed for intraoperative guidance.

### 4.3. Intraoperative Applications

During surgery, AI technologies are enhancing real-time decision support. AR implementation in which digital overlays are projected onto the surgeon’s visual field can guide tumor resection and preservation of key structures. For instance, researchers are developing head-mounted AR display systems that project AI-analyzed data (such as tumor boundaries or vital structures) onto the operative field [49]. A major barrier in soft tissue surgery is the “soft tissue shift,” where the tumor moves once the skin is incised. AI algorithms utilizing deformable registration are solving this by warping preoperative models to fit the intraoperative reality, although these are still investigational. A recent study introduced a framework using both pre-resection external surfaces and post-resection cavities to model specimen thickness, improving Target Registration Error by up to 33% in tongue specimens and improved average target relocation errors from 9.8 mm to 4.8 mm, bringing AR within the realm of clinical safety [7].

Another critical intraoperative need in head and neck cancer surgery is accurate margin assessment. AI is making significant strides here through advanced optical imaging and real-time tissue analysis. Traditional frozen section pathology during surgery is labor-intensive and time-consuming, with accuracy limitations. Hyperspectral Imaging (HSI) captures light across hundreds of spectral bands to differentiate tumor from healthy tissue based on metabolic signatures [8]. Researchers have combined hyperspectral imaging (HSI) with deep learning to differentiate tumor from normal tissue during surgery without any dye or contrast [50]. In this approach, freshly resected specimens were scanned with HSI across many wavelengths and processed by CNNs. AI could then predict tumor presence with 98% accuracy and 93% sensitivity, matching closely with gold-standard histopathology. This kind of label-free, rapid optical analysis can be integrated into the surgical workflow to guide surgeons on where residual tumor might remain at the margins, potentially outperforming the 63% accuracy of frozen sections. An important limitation of the HSI studies cited is that they were performed on ex vivo surgical specimens rather than in real-time intraoperative settings with live tissue. Translation to an intraoperative workflow requires resolution of significant technical challenges, including motion artifact, tissue desiccation, and time constraints of an ongoing operation. These findings are therefore best understood as proof-of-concept data rather than clinical-readiness evidence. Other modalities like fluorescence imaging, Raman spectroscopy, and optical coherence tomography can also be paired with AI algorithms to identify cancerous tissue in real time [51,52,53,54,55,56]. AR can then display these AI-identified cancer areas to the surgeon. As these tools mature, surgeons could have immediate feedback on margin status, enabling more resections with negative margins, as well as real-time, more precise re-excisions.

AI-assisted features are being explored to enhance robotic surgery in head and neck oncology by introducing elements of autonomy, precision, and safety. Traditional robotic systems, such as the da Vinci platform, rely entirely on direct human control; however, newer AI-integrated “cooperative” robotic systems can assist the surgeon in more active and intelligent ways [57]. For instance, AI models can enhance the surgical field by removing “smoke” from electrocautery in real-time or perform “denoising” and color correction to improve visual clarity during deep-cavity procedures. DL models can identify surgical planes and distinguish between healthy tissue and tumors, with some models generating heatmaps of probable cancer locations during surgery [58]. Since current robots lack haptic feedback, AI is also being used to estimate grip force and tissue tension, preventing accidental tissue damage [59].

An underexplored but clinically important intraoperative application domain is AI-assisted reconstructive planning. Reconstruction after oncologic head and neck surgery is inherently complex, requiring surgeons to evaluate defect characteristics, patient comorbidities, vascular anatomy, prior treatment history, and the spectrum of available flap options—from local and regional pedicled flaps to free tissue transfer. Traditionally, this decision-making process relies heavily on surgeon experience and centers on a relatively small set of well-established reconstructive algorithms. AI-based decision support tools have the potential to systematize this process by analyzing defect type, geometry, and patient-specific factors to suggest feasible reconstructive options ranked by predicted outcomes such as flap viability, functional restoration, and donor site morbidity. Rather than replacing the reconstructive surgeon’s judgment, such tools could serve as a cognitive aid, particularly for complex or unusual defects where the optimal reconstructive strategy is less clearly defined. Furthermore, integrating AI-driven reconstructive planning with preoperative imaging and tumor segmentation could eventually allow surgeons to simulate both oncologic resection and reconstruction in a single planning workflow. Furthermore, such planning could be used as a patient counseling and trainee education tool. At present, clinical evidence for AI in reconstructive decision support remains limited, and prospective validation is needed before such tools can be responsibly integrated into routine care.

### 4.4. Postoperative Applications

After initial treatment, AI plays a growing role in predicting patient outcomes and informing surveillance strategies in head and neck oncology. Numerous studies have applied ML algorithms to clinical, pathological, and imaging data to forecast outcomes such as survival, disease recurrence, and treatment complications [9,10,60]. For instance, predictive models have been built to estimate overall survival and disease-free survival based on patterns in patients’ demographics, tumor features, and therapy details [9]. Integrating radiomic features with clinical data improved prediction of 2-year survival in head and neck cancer compared to clinical data alone [61].

Beyond cancer control outcomes, AI can be used to foresee postoperative complications and resource needs. Models have been created to predict wound complications, gastrostomy dependence, free flap failure, or postoperative length of hospital stay based on pre-surgical factors, intraoperative data, and tumor stage [62,63,64]. Most published complication prediction models in head and neck surgery have been developed and validated within single institutions, limiting their generalizability. External performance is rarely reported, and the clinical threshold at which a model’s predicted risk would change management has not been defined. Prospective implementation studies that embed these tools into preoperative decision-making and measure their impact on patient outcomes (rather than simply their predictive accuracy) remain an important unmet need. Integration of these predictive capabilities into clinical workflows may allow for targeted interventions for vulnerable populations, including but not limited to patient education, or enhanced rehabilitation and nursing services. AI chatbots also hold promise to aid patients in their postoperative recovery, potentially augmenting patient education while decreasing workload on healthcare teams [60].

The postoperative phase in head and neck oncology also involves long-term surveillance for recurrence. Here, AI is augmenting traditional follow-up by analyzing subtle signals earlier than humans might detect. One frontier is liquid biopsy monitoring: AI can interpret trends in circulating tumor DNA or other blood biomarkers over time to signal minimal residual disease and impending recurrence, although prospective evidence linking AI-interpreted predictions to improved survival does not yet exist [65]. Patient-generated health data can also play a role in postoperative monitoring. For instance, AI-assisted analysis of voice recordings or swallowing metrics post-laryngectomy or oropharyngeal surgery may detect functional deterioration indicating a tumor recurrence or second primary [66]. By continuously learning from each patient’s post-treatment profile, AI systems may improve lead time detection of cancer recurrence for possibly earlier intervention.

## 5. Challenges

**Figure 4:**
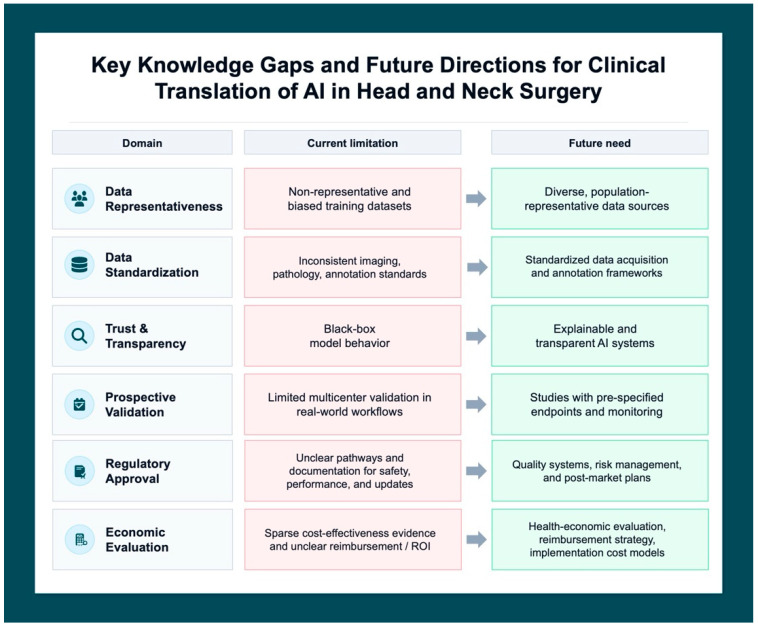
Key Knowledge Gaps and Future Directions for the Clinical Translation of Artificial Intelligence (AI) in Head and Neck Surgery. The framework identifies six primary domains. Red panels indicate current systemic bottlenecks, while green panels denote the requisite advancements for successful clinical implementation.

For decades, the fundamental tenets of head and neck surgical oncology have remained consistent: complete oncologic resection, management of the cervical lymphatics, and functional reconstruction. While technological advances have refined these tasks, the cognitive burden of decision-making and the sensory limitations of the human operator have remained constant constraints. AI promises augmentation for the head and neck surgical oncologist; however, several barriers must be addressed before routine adoption (Figure 4).

### 5.1. Data Quality, Representativeness, and Bias

AI systems are only as reliable as the data on which they are trained [67]. Imaging, pathology, and clinical data require accurate annotations and formatting (e.g., imaging standards, standardized pathology labels) to allow effective training and comparison of algorithms. Bias remains a central concern, as training datasets may underrepresent certain racial, socioeconomic, or geographic populations, which could lead to AI algorithms propagating inequities in prognostic estimation or treatment recommendations [68].

Harmonized, representative datasets with intentional bias mitigation are critical [69]. Furthermore, federated learning—where models are trained across institutions without transferring raw data—offer one pathway to improve generalizability while preserving patient privacy [70]. However, such approaches require substantial technical infrastructure and coordination.

An additional source of heterogeneity is the diversity of imaging protocols across institutions, including variation in CT slice thickness, contrast timing, MRI field strength, and endoscopy equipment. Pathology annotation is similarly institution-dependent, with variability in reporting standards for nodal levels, margin definitions, and tumor grade. These sources of data heterogeneity compound the challenge of building generalizable AI models and must be explicitly addressed in future multicenter dataset curation efforts.

### 5.2. External Validation and Clinical Utility

Many AI models report strong discrimination in retrospective application to internal datasets, but performance declines in external validation cohorts. Prospective validation in real-world clinical settings, particularly in a randomized fashion, remains limited.

Critically, AI tools have not yet demonstrated improvement in outcomes, such as survival, margin status, complications reduction, or quality of life. The transition from proof-of-concept to demonstrated clinical value will require prospective implementation studies, careful assessment of workflow impact, and acceptability to the physicians and/or the patients who use these AI tools.

### 5.3. Workflow Integration and Surgeon Trust

Even technically robust and validated models may fail if poorly integrated into the clinical workflow. Tools at the point-of-care, with accessible user interface design, and interpretable and actionable outputs are most likely to gain traction with physicians.

Clinicians often express mistrust or lack of understanding of AI “black boxes.” If an algorithm cannot explain its reasoning, it is hard for surgical oncologists to fully trust its recommendations over their own expertise. Currently, many advocate for a “human-in-the-loop” model combined with transparent, interpretable outputs (e.g., heatmaps highlighting image regions of concerns, confidence intervals, and traceable source) [71].

### 5.4. Regulatory and Medicolegal Considerations

AI systems deployed in clinical care are increasingly regulated as medical devices, yet jurisprudence regarding liability in AI-assisted patient care is still evolving [72].

No consensus framework has emerged for liability in the setting of AI-related error. Clinicians, developers, and healthcare institutions may each bear responsibility [73]. Liability may depend in part on the degree of algorithmic autonomy, with clinicians likely assuming greater liability when AI is used in a decision-support role, and liability shifting toward developers or institutions with increasing algorithmic independence [74]. As AI tools become more widely validated, the standard of care may evolve to incorporate their use. Adoption can be risky. Failure to adopt improved technologies and inappropriate reliance on flawed outputs may both expose surgeons to liability.

The “black box” nature of many AI systems adds further complexity to liability attribution, as limited explainability may hinder a surgeon’s ability to justify AI-influenced decisions, particularly in high-stakes settings such as tumor margin assessment or treatment de-escalation [75]. This raises important considerations for informed consent, as physicians may be unable to fully explain the basis of AI-generated recommendations, potentially limiting patient autonomy [76]. While the use of AI in clinical decision-making should likely be disclosed to patients, the appropriate extent of disclosure remains uncertain given concerns that overemphasis on AI involvement may undermine patient trust when these tools function primarily as adjuncts to clinician judgment [76].

Additional concerns arise in the use of patient data for AI development, where evolving regulatory frameworks, challenges in obtaining meaningful consent, and risks of data breaches necessitate robust data governance, transparency, and privacy protections [77]. Clear documentation of decision-making rationale, incorporation of human oversight, and implementation of audit trails and post-deployment surveillance will be essential to mitigate medicolegal risk as AI becomes increasingly integrated into head and neck surgical oncology.

### 5.5. Economic and Operational Realities

Development, validation, and maintenance of AI systems require significant investment in infrastructure, data engineering, cybersecurity, and personnel expertise [78,79,80]. Cost-effectiveness analyses remain limited, particularly in surgical oncology.

## 6. Conclusions

This review provides a comprehensive synthesis of emerging applications of artificial intelligence across head and neck surgical oncology, organized along the continuum of care from preoperative planning to intraoperative decision-making and postoperative outcomes and surveillance. Importantly, this work aims to bridge the gap between technical development and clinical practice by equipping surgeons with a framework to understand, evaluate, and responsibly integrate AI tools into patient care. Across these domains, we highlight both the breadth of current applications and the variability in performance, validation, and clinical readiness, while identifying key barriers to clinical translation.

As clinical practice becomes increasingly integrated with AI systems, rigorous validation and oversight will be required to establish where AI truly adds value and where human judgment remains irreplaceable. AI will not replace surgeons, but surgeons who understand and appropriately deploy AI tools will likely outperform those who do not. As with prior technological shifts in head and neck oncology—from endoscopy to microvascular reconstruction to transoral robotics—the surgeons who critically engage with these tools will shape how they are safely integrated into practice.

## Figures and Tables

**Table 1:** Summary of Select Studies Evaluating AI Applications in Head and Neck Surgical Oncology.

Author (Year)	Application	Methods	Key Metric	Validation	Limitations
Preoperative					
Lan et al. (2024) [2]	Occult cervical LN metastasis detection (MRI-based DL + radiomics)	CNN on MRI for oral/oropharyngeal SCC; training + external cohort	AUC 0.92 (training), AUC 0.72 (external validation)	External	Large performance drop across cohorts; retrospective design; lacks prospective validation
Dayan et al. (2026) [3]	Imaging-based ENE detection + outcome prediction (HPV+ OPC)	DL model on pretreatment CT; HPV+ OPC	Outperformed radiologist ENE detection; improved outcome stratification	Internal cross validation	Single cancer subtype (HPV+ OPC); CT-only input; institutional protocol variation
Kann et al. (2023) [4]	CT-based DL screening for ENE in HPV+ OPC (de-escalation trial context)	DL algorithm evaluated on multicenter RCT patient data	Validated sensitivity/specificity for ENE in multicenter trial setting	Multicenter RCT data	Prospective integration into clinical workflow not yet assessed; HPV+ only
Klein et al. (2023) [5]	DL prediction of HPV status in OPC from H&E histology slides	CNN on whole-slide H&E images; multi-institutional cohort	High AUC for HPV status classification directly from H&E	Multi-institutional; internal cross validation	OPC-specific; requires high-quality slide digitization; not yet adopted clinically
Unlu et al. (2025) [6]	LLM-assisted clinical trial prescreening vs. manual chart review	RCT comparing AI vs. manual eligibility screening	Comparable accuracy to manual review; significantly reduced time burden	RCT	General oncology (not head and neck-specific); may miss nuanced eligibility criteria
Intraoperative					
Yang et al. (2026) [7]	AR-guided tumor resection with deformable registration (tongue)	Pre/post-resection surface registration framework; tongue specimens	TRE improved by 33%; average error reduced from 9.8 mm to 4.8 mm	Internal cross validation	Investigational; not validated in live surgery; limited anatomic sites tested
Halicek et al. (2019) [8]	HSI + DL for intraoperative margin assessment	HSI + CNN on 102 surgical specimens (HNSCC)	98% accuracy, 93% sensitivity vs. histopathology gold standard	Internal cross validation	Ex vivo specimens only; no real-time intraoperative trial; limited to HNSCC
Postoperative					
Karadaghy et al. (2019) [9]	ML model for overall survival prediction in oral SCC	Random forest + clinical/pathologic features; SEER + institutional data	AUC 0.77 for 5-year survival prediction	Internal cross validation	Retrospective; limited granularity on treatment details; no functional outcomes
Formeister et al. (2020) [10]	ML for predicting complications in HN microvascular free tissue transfer	Logistic regression + random forest on institutional free flap cohort	Improved complication prediction vs. traditional scoring tools	Internal cross validation	Single institution; small sample; models not externally validated
You et al. (2025) [11]	Ambient documentation AI: impact on documentation burden and burnout	Large multi-site survey of clinicians using ambient AI-drafted notes	Reduced perceived documentation burden; improved well-being scores	Multi-site survey	Self-report only; no objective outcome measures; no head and neck-specific subgroup

Abbreviations: AI, artificial intelligence; AUC, area under the curve; CNN, convolutional neural network; CT, computed tomography; DL, deep learning; ENE, extranodal extension; H&E, hematoxylin and eosin; HNSCC, head and neck squamous cell carcinoma; HPV, human papillomavirus; HIS, hyperspectral imaging; LLM, large language model; LN, lymph node; ML, machine learning; MRI, magnetic resonance imaging; OPC, oropharyngeal cancer; RCT, randomized controlled trial; SCC, squamous cell carcinoma; SEER, Surveillance Epidemiology and End Results Program; TRE, target registration error.

## Data Availability

No new data were created or analyzed in this study. Data sharing is not applicable to this article.

## Abbreviations

The following abbreviations are used in this manuscript:

AI	Artificial intelligence
ANN	Artificial neural networks
AR	Augmented reality
AUC	Area under the curve
CNN	Convolutional neural networks
DL	Deep learning
EHR	Electronic health record
ENE	Extranodal extension
GAN	Generative adversarial networks
HIPAA	Health Insurance Portability and Accountability Act
HSI	Hyperspectral imaging
LLM	Large language model
ML	Machine learning
NLP	Natural language processing
PHI	Protected health information
RAG	Retrieval-augmented generation
